# Management of a Nipple Injury Causing Nipple Bifurcation and Infection

**DOI:** 10.7759/cureus.68833

**Published:** 2024-09-06

**Authors:** Nicholas J Carter

**Affiliations:** 1 Department of Family and Community Medicine, Evans Army Community Hospital, Fort Carson, USA

**Keywords:** austere nipple bifurcation, austere nipple infection, austere nipple injury, nipple bifurcation, nipple flap, nipple infection, nipple injury

## Abstract

Military physicians working in resource-constrained environments, including reduced access to care, must evaluate for any circumstances that would necessitate an aeromedical evacuation to a higher level of care; this is particularly important in foreign countries. Due to these circumstances, military physicians must be resourceful while maintaining expertise to stabilize and treat any medical issue whether or not evacuation is requested. A 26-year-old female requested urgent care evaluation after she endured an accidental traumatic nipple stud removal at an austere military base located in Egypt. Upon initial evaluation at the base clinic, a left nipple bifurcation was identified. The case was complicated by a subsequent spontaneously draining bacterial infection at the base of the bifurcation, which resolved without further complication following antibiotic course and daily monitoring. Furthermore, adhesive strips were applied daily to approximate the nipple, which was critical to the restoration of gross anatomy. As such, the nipple bifurcation was held in place utilizing adhesive strips, thereby producing counterforce to facilitate healing by primary intention for two weeks resulting in a favorable cosmetic outcome. Ultimately, the patient's clinical course resulted in a well-healed and well-approximated linear scar on her left nipple. Long-term distal milk duct function is unable to be assessed outside of lactation periods or advanced imaging, but given the location and extent of the injury, a plastic surgery evaluation and imaging were deferred. This case report outlines the management considerations, literature search, and treatment course of a rare, unreported injury type in an austere, military environment along with consideration of future research.

## Introduction

Despite nipple piercings becoming more popular in recent times [1], accidental traumatic nipple injuries are not well documented. Most literature focuses on nipple trauma related to feeding neonates in the post-partum time frame, which is the most common cause of nipple pain and trauma [2]. There are no cases describing a nipple bifurcation. There are no cases describing management or antimicrobial considerations following a traumatic removal of a nipple piercing. Nipple infections are well-documented in the literature, but typically in the presence of breastfeeding or in the immediate period following nipple piercing placement [3]. Furthermore, there was no research that discussed antimicrobial pathogenic species nor antimicrobial coverage for antibiotic therapy in traumatic nipple piercing removal. This paper aims to describe the clinical course and discuss the management consideration of an undocumented injury caused by a rare traumatic etiology.

## Case presentation

**Figure 1:**
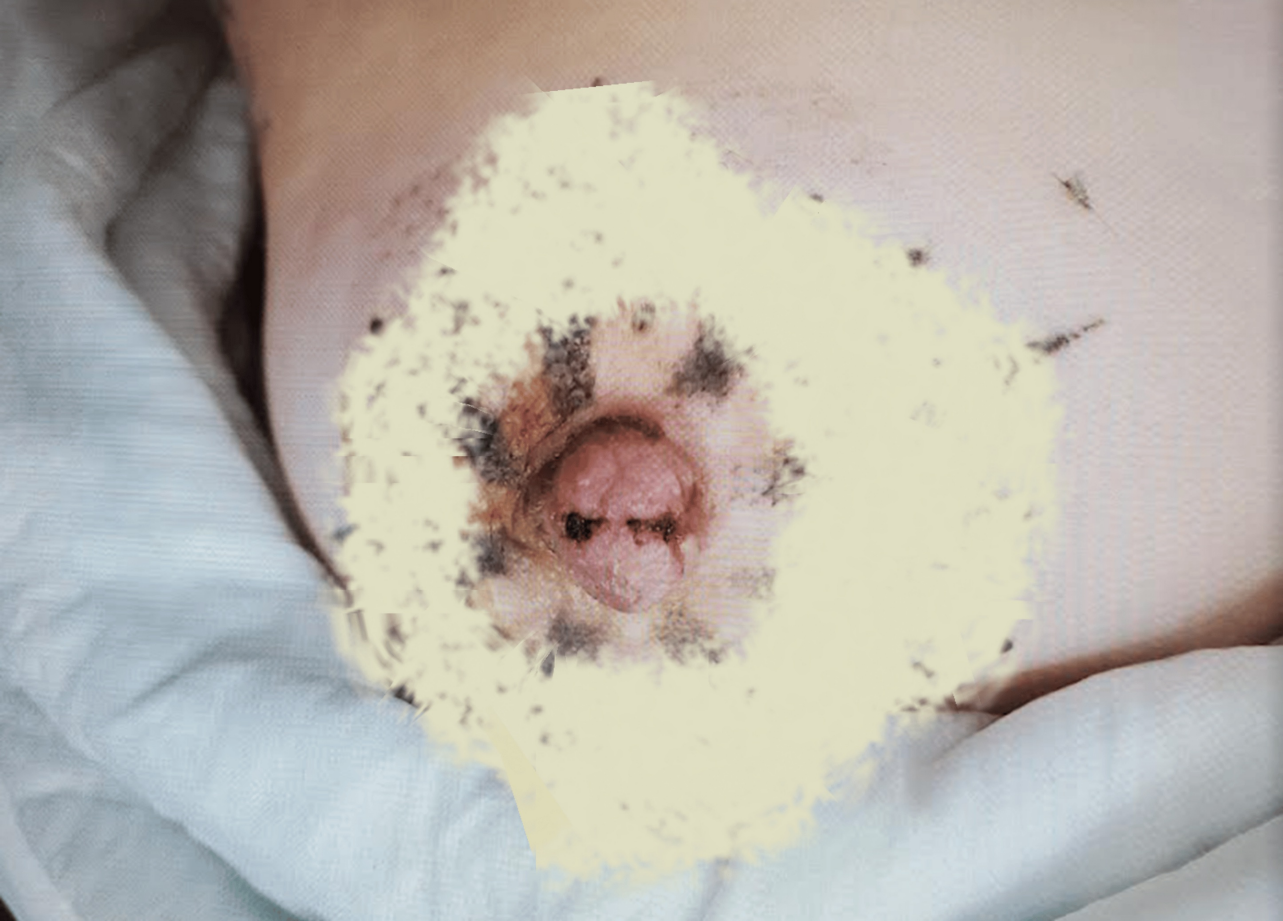
Injured nipple Fibrous clot weakly adhering nipple flaps. Image altered to obscure identifying tattoos.

**Figure 2:**
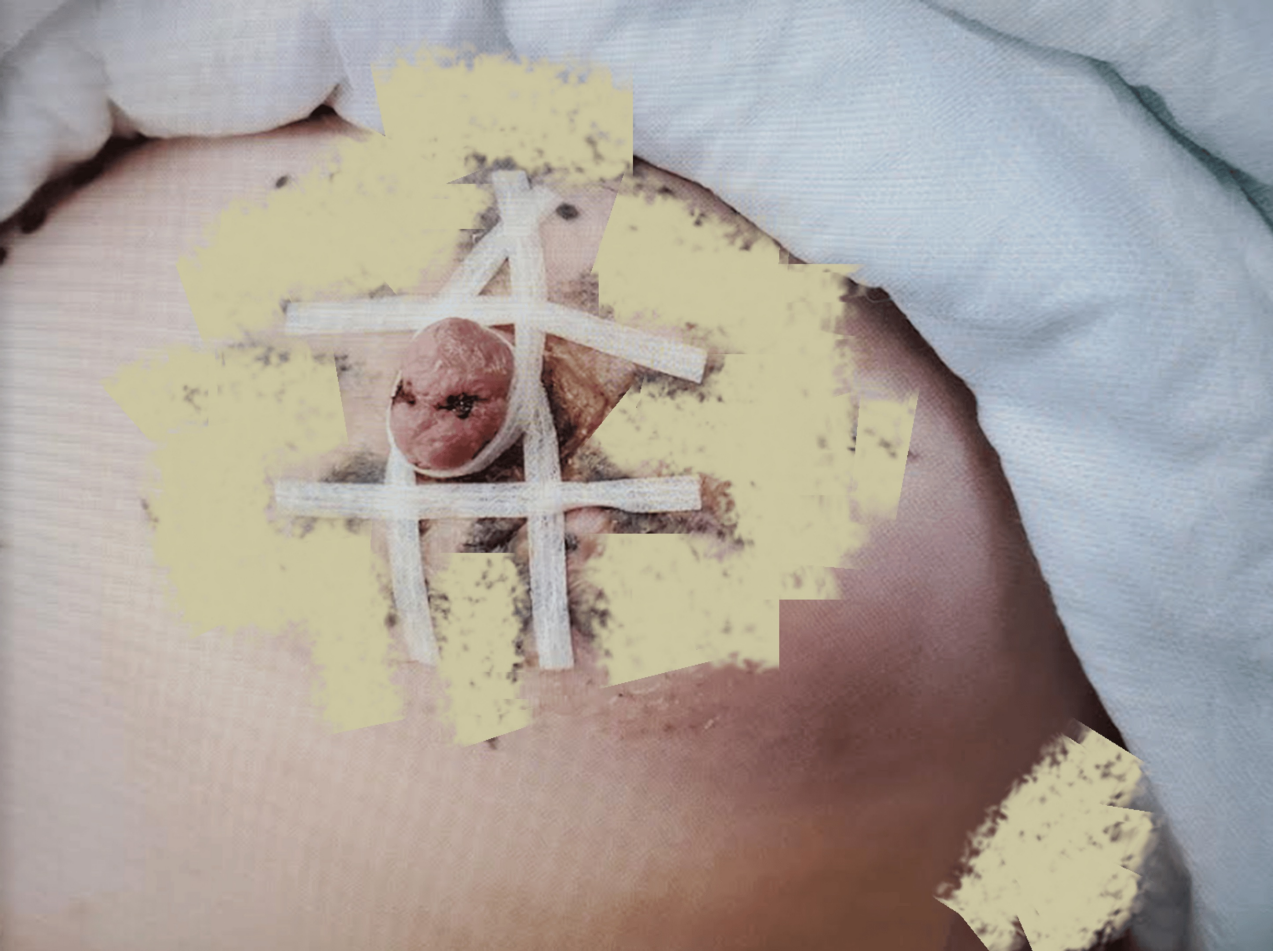
Adhesive strip placement View one: Adhesive strips placed to provide nipple flap counterforce, facilitating healing by primary intention. The image was altered to obscure identifying tattoos.

**Figure 3:**
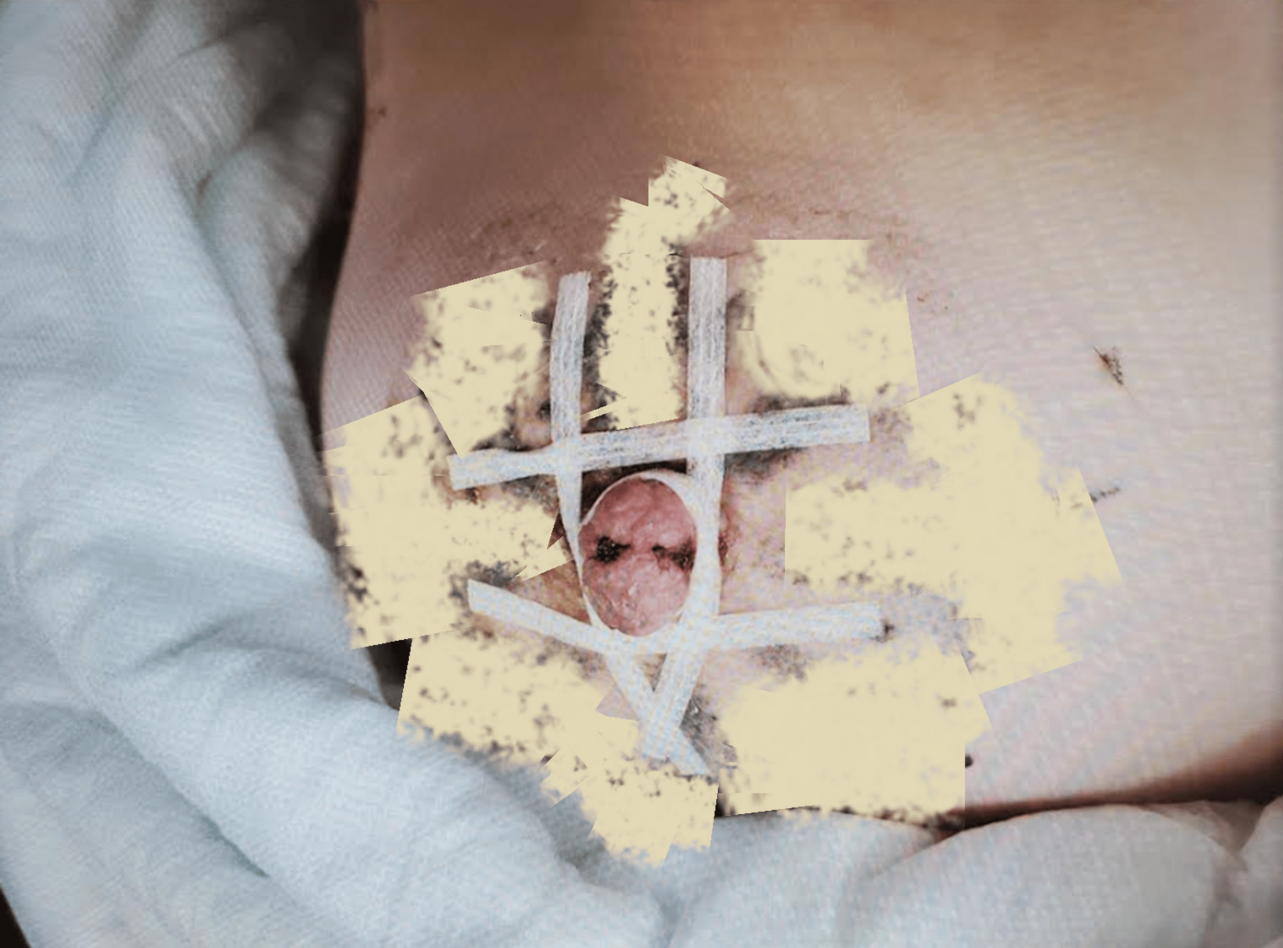
Adhesive strip placement View two: Adhesive strips placed to provide nipple flap counterforce, facilitating healing by primary intention. The image was altered to obscure identifying tattoos.

A 26-year-old non-gravid female utilized a United States military clinic's "after-hours" on-call urgent care service at an austere base located in Sharm El Sheikh, Egypt. She was evaluated and found to have endured an accidental traumatic left nipple stud removal after the stud was caught on a shower door resulting in a left linear nipple bifurcation to 1/8th of an inch from the base of the nipple approximately eight hours prior to evaluation. At the time of presentation, a fibrous clot held the nipple flaps together (Figure 1). While the injury was painful, the patient tolerated the examination well. The nipple was cleaned with flap deviation occurring when the fibrous clot was dissolved during irrigation and similarly the nipple bisected when she conducted personal hygiene such as taking a shower; the bifurcation almost perfectly bisected the nipple with the vertical nipple flaps noted to laterally deviate from the center. This necessitated a reapproximation of the gross nipple anatomy utilizing adhesive strips. The adhesive strips were placed in such a way as to secure the nipple during the healing process. The adhesive strip placement was intentional where a "U" shape with both tails on one side of the nipple, and the center on the strip on the opposite nipple flap was placed to provide counterforce. A second strip was used to place the "U" shape on the contralateral side to place counterforce to the first in a similar fashion thereby reapproximating the nipple to normal anatomical location and promoting healing by primary intention. Once the gross anatomy of the nipple was secured, secondary adhesive strips were placed across the tails to anchor the strips, minimizing the chance the primary strips would fail (Figure 2 and Figure 3). Once the wound was tended, a literature search was conducted to aid medical management such as infection occurrence rate, antimicrobial coverage, fibrosis risk to nipple duct function, and/or cosmetic outcome requiring the need for nipple reconstruction.

While some of these considerations would necessitate an aeromedical evacuation to a higher level of care such as a plastic surgery consultation, a patient-centered discussion of the risks versus benefits of evacuation with a focus on evaluation by a plastic surgeon was held. This discussion led to the decision that the patient did not require evacuation to a higher level of care by the treating physician who simultaneously served as the clinic's Chief Medical Officer/Officer in Charge, the position of the clinic's evacuation request authority. Instead, the patient would follow up daily for one week followed by every other day thereafter until the nipple healed or if a complication developed outside of the medical scope of the treating physician in that environment and associated resources. The following day, the patient developed increased pain, tactile warmth, and purulent drainage, which were all found at the base of the nipple; no fluctuation was noted. An ultrasound was utilized to evaluate the soft tissue at the base of the nipple. A small, circular hypoechoic pocket measuring 0.25 centimeters was identified at the base of the nipple that remained superficial, not requiring incision and drainage. There was no evidence of sinus tract formation or secondary pocket. An unspecified bacterial skin infection was suspected.

**Figure 4:**
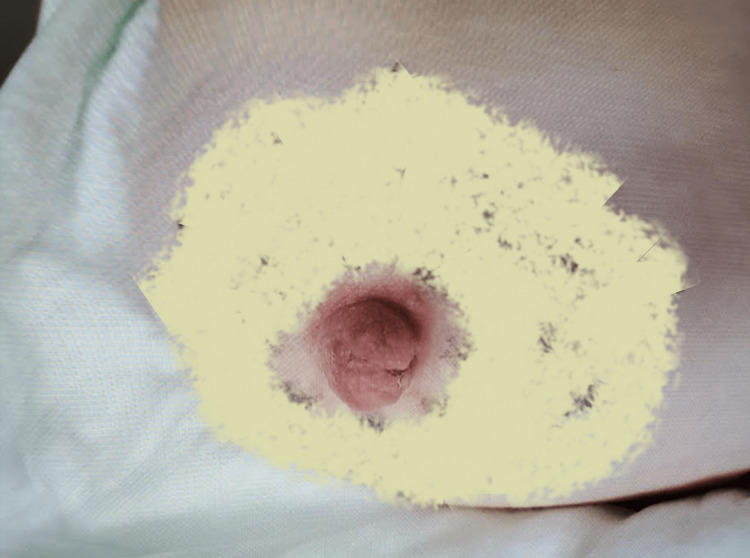
Healed nipple View one: Injured nipple two weeks after presentation. The image was altered to obscure identifying tattoos.

**Figure 5:**
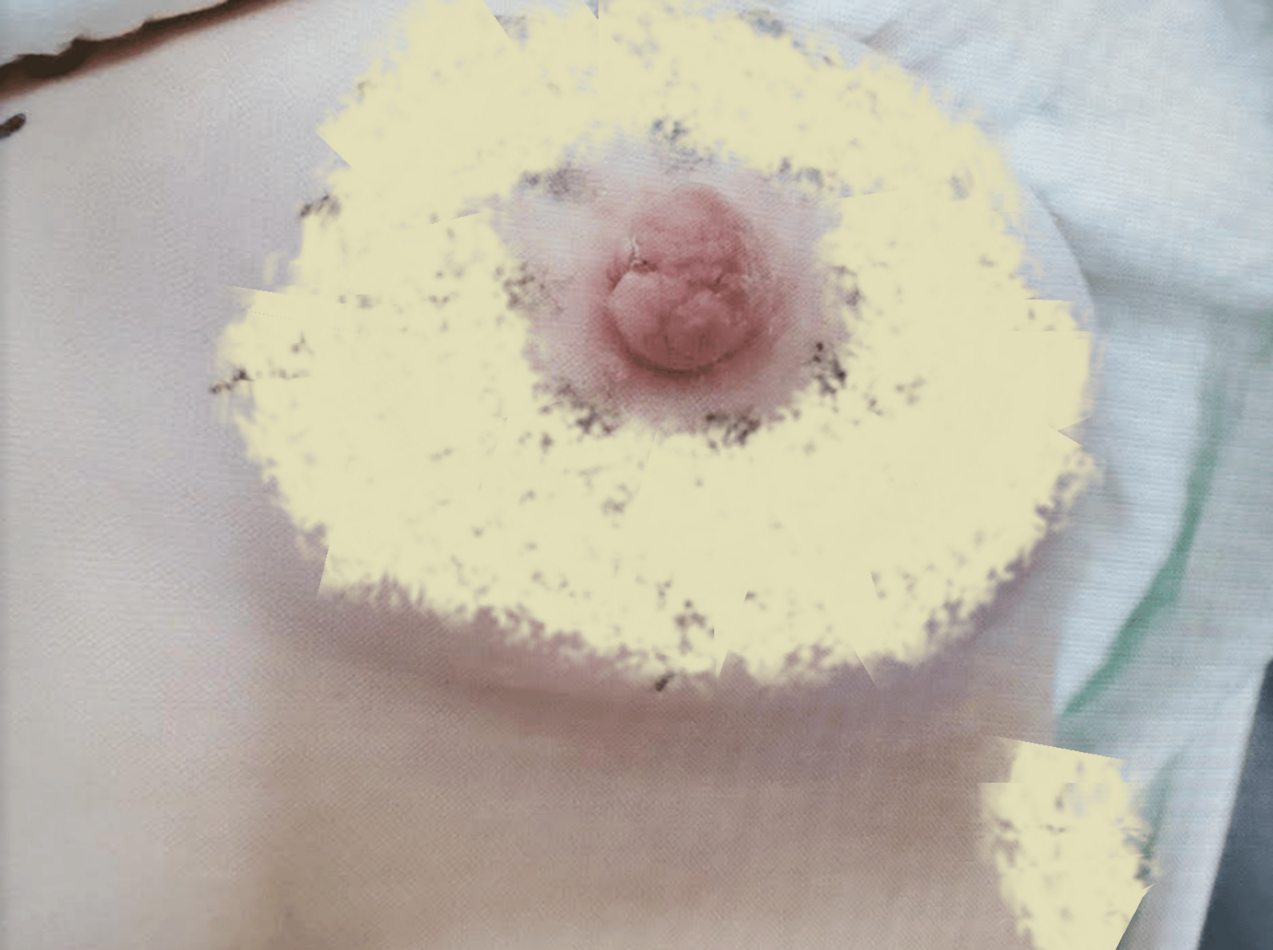
Healed nipple View two: Injured nipple two weeks after presentation. The image was altered to obscure identifying tattoos.

Reviewing the previously discussed literature review, there was literature for antimicrobial coverage targeting specific microbial species in patients with nipple piercings, but only in the setting directly following piercing placement; there was no research found regarding nipple infection antibiotic coverage following traumatic injury nor guidance regarding traumatic nipple bifurcation management. As such, a first-line medication, sulfamethoxezole-trimethroprim, 160 mg/800 mg, respectively, was prescribed to clear the infection due to the presumption of pathogens typical of dermatological infection, such as *Staphylococcus* and *Streptococcus* species, with particular focus its antimicrobial properties toward methicillin-resistant *Staphylococcus aureus* (MRSA) given the remote environment of the clinic. The patient was instructed to take two double-strength tablets by mouth daily for seven days. She was allowed to recover in her quarters continuing daily follow-up. The patient's infectious symptoms improved significantly within 48 hours with a daily decrease in pain, nipple drainage, and tactile warmth. The patient continued to follow up daily for wound management. The adhesive strips were removed and replaced daily. By day-of-injury five, the patient was noted to have a resolution of her infectious signs and symptoms. The remainder of her clinical course remained uncomplicated. Two weeks following her initial evaluation, the nipple displayed a well-healed linear fibrotic scar with successful gross restoration of the nipple anatomy and favorable cosmetic outcome (Figure 4 and Figure 5).

## Discussion

This case report describes the management considerations of a rare injury, the traumatic nipple bifurcation that was complicated by bacterial infection. The particular focus of the patient’s care was to restore normal anatomy with favorable cosmetic results and consider the effect on the lactiferous ducts that travel adjacently and longitudinally from the proximal nipple with orifices terminating at the distal nipple. Abnormal healing could result in the inability of that nipple to deliver milk produced in mamillary lobules following a potential future pregnancy. Therefore, adhesive strips were deliberately fashioned around the nipple to provide a counterforce to facilitate healing by primary intention and minimize the risk of abnormal wound healing. The identification of the bacterial infection, daily wound management, and healing by primary intention prevented the disfigurement and derangement of normal nipple anatomy. Physiological reasoning implies that the restoration of anatomy and minimizing fibrosis positively restored some percentage of functionality of breast milk delivery that would have otherwise been lost. Garbin et al. stated that nipple piercing does not negatively impact milk supply [4]. However, scar tissue, whether accidental and secondary to trauma as in this case or controlled such as conducting nipple piercing, may decrease functional lactation due to nipple duct fibrosis [5]. It is important to note that the specific change in functionality from the pre- and post-injurious state is unknown.

## Conclusions

This case illustrates a nipple bifurcation from the onset of injury through the healing process, including bacterial infection and daily wound care. The nipple would have been disfigured if healing by primary intention was not accomplished by counterforce adhesive strips; the "U" pattern utilized on each side of the nipple with contralateral anchoring was highly successful. As such, distal milk duct function and future potential newborn breastfeeding are minimally impacted by the restoration of gross anatomy. Finally, restoration of gross anatomy minimizes the impact of symptoms and irritation when wearing clothing and apparel, including military gear. In conclusion, this case highlights the importance of clinical management considerations of rare injuries in resource-limited environments. It is hopeful that this case report will facilitate others who encounter a nipple injury, particularly bifurcation, in their medical management. Further research should be focused on milk duct function in these injury types compared to normal and the impact on newborn breastfeeding in addition to antimicrobial considerations of accidental traumatic nipple injuries involving nipple piercings.
